# Abnormal Spatial Patterns of Intrinsic Brain Activity in Osteonecrosis of the Femoral Head: A Resting-State Functional Magnetic Resonance Imaging Study

**DOI:** 10.3389/fnhum.2020.551470

**Published:** 2020-09-17

**Authors:** Shengyi Feng, Bo Li, Gang Li, Xuyun Hua, Bo Zhu, Xuejia Li, Wenting Lu, Jianguang Xu

**Affiliations:** ^1^Center of Traumatology and Orthopedics, Yueyang Hospital of Integrated Traditional Chinese and Western Medicine, Shanghai University of Traditional Chinese Medicine, Shanghai, China; ^2^Department of Orthopedics, Affiliated Hospital of Shandong University of Traditional Chinese Medicine, Shanghai, China; ^3^Quyang Community Health Service Center of Hongkou District, Shanghai, China; ^4^School of Rehabilitation Science, Shanghai University of Traditional Chinese Medicine, Shanghai, China

**Keywords:** osteonecrosis of the femoral head, magnetic resonance imaging, spatial patterns, spontaneous brain activity, amplitude of low-frequency fluctuations, Harris hip scores, sensorimotor network, osteonecrosis

## Abstract

**Objective**: Osteonecrosis of the femoral head (ONFH) is a common condition that is encountered in clinical practice, and yet, little is known about its characteristics and manifestations in the brain. Therefore, in this study, we aimed to use resting-state functional magnetic resonance imaging (rs-fMRI) to investigate the spatial patterns of spontaneous brain activity in the brain of ONFH patients.

**Methods**: The study included ONFH patients and healthy controls. The pattern of intrinsic brain activity was measured by examining the amplitude of low-frequency fluctuations (ALFF) of blood oxygen level-dependent signals using rs-fMRI. Meanwhile, we also used Harris hip scores to evaluate the functional performance of ONFH patients and healthy controls.

**Result**: Ten ONFH patients and 10 health controls were investigated. We found global ALFF differences between the two groups throughout the occipital, parietal, frontal, prefrontal, and temporal cortices. In the ONFH patients, altered brain activity was found in the brain regions in the sensorimotor network, pain-related network, and emotion and cognition network. The results of the correlation investigations also demonstrated that the regions with ALFF changes had significant correlations with the functional performance of the patients evaluated by Harris hip scores.

**Conclusions**: Our study has revealed the abnormal pattern of brain activity in ONFH patients, and our findings could be used to aid in understanding the mechanisms behind the gait abnormality and intractable pain associated with ONFH at the central level.

## Introduction

Osteonecrosis of the femoral head (ONFH) is caused by impaired or interrupted vascular supply, and it may occur in individuals of all ages (Sultan et al., 2019). Subsequently, it may lead to the necrotization of the osteocytes. ONFH may originate from both traumatic and non-traumatic aetiologies. As the area of necrosis increases, the manifestations of ONFH progress to structural modifications and the collapse of the femoral head. As a consequence, ONFH may be accompanied by varying levels of pain and the functional disability of the hip joint. Among all of its symptoms, pain is usually the most significant and sustained, especially in advanced-stage ONFH [Association Research Circulation Osseous (ARCO) III° and IV°; Pyda et al., 2015; Kuroda et al., 2016].

Nowadays, owing to the advances in brain imaging techniques, a larger number of researchers are focusing on the central mechanism of arthritic disorders. The pathophysiologies of several motor system diseases have been investigated using functional magnetic resonance imaging (fMRI) techniques. In patients with osteoarthritis, Chen et al. (2015) reported that acupuncture might relieve knee osteoarthritis-related pain by modulating the functional connectivity between the right frontoparietal network, executive control network, and descending pain modulatory pathway. Within the networks, the rostral anterior cingulate cortex and medial prefrontal cortex were found to exhibit enhanced functional connectivity (Chen et al., 2015). A study based on the moxibustion treatment of knee osteoarthritis demonstrated increased fractional amplitude of low-frequency fluctuation (fALFF) values in the bilateral cerebrum, extra-nucleus, left cerebellum, and white matter. Simultaneously, the fALFF values of the precentral gyrus (PreCG), frontal lobe, and occipital lobe were found to be decreased (Xie et al., 2013). In rheumatoid arthritis patients, Flodin et al. (2016) reported that the supplementary motor areas, midcingulate cortex, and primary sensorimotor cortex exhibited enhanced functional connectivity. A recent multi-modal MRI study revealed that positive connections between the inferior parietal lobule, medial prefrontal cortex, and multiple brain networks, as well as reductions in inferior parietal lobule Gray Matter (GM), could be monitored to predict the development of fatigue, pain, and cognitive dysfunction in rheumatoid arthritis patients (Schrepf et al., 2018). Further, a neuroimaging study has reported that patients with chronic osteoarthritis pain exhibit significantly higher levels of anticorrelation between the right anterior insula and default mode network regions, thereby suggesting an altered brain state at rest characterised by the increased inhibitory effect of the salience network on the default mode network (Ryan et al., 2018).

Most studies focus on the pain-related symptoms following the onset of osteoarthritis and rheumatoid arthritis. However, in patients suffering from ONFH, gait abnormality is also an important symptom that may cause a dramatic reorganization in the motor-related brain networks. Thus, it is assumed that multiple brain networks may be involved in the adaptive plasticity following the development of ONFH and that resting-state fMRI (rs-fMRI) could be an effective modality for investigating the corresponding changes in the whole brain. Despite this, little is known about the characteristics and manifestations of ONFH in the brain.

Therefore, in the present study, we aimed to investigate the changes in intrinsic or spontaneous brain activity in ONFH patients *via* the use of rs-fMRI to examine the regional amplitude of low-frequency fluctuations (ALFF) across the bilateral hemispheres.

## Materials and Methods

### Subjects

This study was approved by the Medical Research Ethics Committee of the authors’ affiliated institutions and was registered in the national clinical trial registry. Twenty right-handed subjects (10 ONFH patients and 10 healthy controls) participated in this study after providing written informed consent. The participants with ONFH were recruited from among patients who had consulted orthopedic clinics at the authors’ affiliated institutions for hip pain. The healthy controls were recruited from the local community by advertisements.

The inclusion criteria for ONFH patients were as follows: (1) had been diagnosed as ONFH following the 2015 Guideline for Diagnostic and Treatment of ONFH (Li, 2015); (2) were the first time to suffer from ONFH; (3) had no previous treatment of medication; and (4) underwent a complete physical and radiological examination, standard laboratory tests, and an extensive number of neuropsychological assessments.

The criteria for the selection of the healthy controls were as follows: (1) no neurological or psychiatric disorders, such as stroke, depression, or epilepsy; (2) no neurological deficiencies, such as visual or hearing loss; (3) no abnormal findings, such as infarction or focal lesion in conventional brain MRI; (4) no cognitive complaints; and (5) Mini-Mental State Examination (MMSE) scores of 28 or higher.

The exclusion criteria for the patients with ONFH were as follows: (1) presence of neurological or psychiatric disorders, such as stroke, depression, or epilepsy; (2) presence of neurological deficiencies, such as visual or hearing loss; (3) presence of abnormal findings, such as infarction or focal lesion, in conventional brain MRI; (4) cognitive complaints; (5) MMSE scores of less than 28; and (6) the inability to tolerate fMRI scans.

The clinical and demographic data of the 20 participants are presented in Table 1. There were no significant differences between the two groups in terms of sex, age, MMSE scores, and Harris hip scores.

**Table 1:** Demographic data and clinical characteristics of the ONFH patients and healthy controls.

Characteristic	ONFH	Controls	*P*-value
Number (*n*)	10	10	/
Male/female (*n*)	6/4	4/6	0.371^#^
Age (mean ± SD, years)	54.30 ± 19.00	55.7 ± 10.72	0.842^##^
MMSE scores	28.90 ± 2.85	29.60 ± 0.84	0.465^##^
Harris scores	73.10 ± 5.19	97.40 ± 3.53	<0.001^##^

*ONFH, Osteonecrosis of the femoral head; MMSE, Mini-Mental State Examination; SD, standard deviation; ^#^indicates that P-values for sex distribution between the groups were obtained by a chi-square test; ^##^indicates that P-values were obtained by a two-tailed *t*-test*.

### Data Acquisition

The MRI data were acquired by scans performed with a Siemens Verio 3 Tesla scanner (Siemens, Erlangen, Germany). Head-huggers and earplugs were used to limit head motion and reduce the volume of noise produced by the MRI scanner. The subjects were instructed to relax, hold still, keep their eyes closed, keep awake, and not think about anything in particular. Functional images were captured axially using an echo-planar imaging (EPI) sequence with the following settings: a repetition time of 3,000 ms, echo time of 30 ms, a flip angle of 90°, a field of view of 24 cm, a resolution of 64 × 64 matrices, 43 slices with a thickness of 3 mm, a voxel size of 3.75 × 3.75 × 3 mm^3^, and bandwidth of 2,232 Hz/pixel. The scan lasted for 600 s. All the subjects had not fallen asleep based on the responses in a simple questionnaire filled after the scan. Three-dimensional T1-weighted magnetization-prepared rapid gradient echo (MPRAGE) sagittal images were captured using the following parameters: a repetition time of 1,900 ms, echo time of 2.93 ms, an inversion time of 900 ms, a flip angle of 9°, a resolution of a 256 × 256 matrix, 160 slices with a thickness of 1.0 mm, and a voxel size of 1 × 1 × 1 mm^3^.

### Data Preprocessing

In this study, fMRI image data processing was carried out using the Data Processing Assistant for rsfMRI (DPARSF; Yan et al., 2016); this toolbox is based on Statistical Parametric Mapping 8 (SPM8^1^) and is run in MATLAB R2014a (MathWorks Inc., Natick, MA, USA). The preprocessing steps were based on a recent mainstream protocol (Wang et al., 2011): the first 10 volumes of images were discarded owing to signal equilibrium and to allow the participants to adapt to the noise produced by the MRI scanner. All the slices of the remaining 190 volumes were corrected for different signal acquisition times by shifting the signal measured in each slice relative to the acquisition time of the slice acquired in the middle of each repetition time. Subsequently, to minimize the confounding of head motion, we utilized the Friston 24-parameter model to regress out head motion effects (Friston et al., 1996). The Friston 24-parameter model (i.e., six head motion parameters, six head motion parameters one time-point before, and the 12 corresponding squared items) was chosen based on prior work that higher-order models remove head motion effects better (Satterthwaite et al., 2013; Mistry et al., 2016). The motion-corrected functional volumes were spatially normalized to the EPI template space and resampled to 3 mm isotropic voxels using the normalization parameters estimated and recorded during unified segmentation. Following this, the functional images were spatially smoothed with a 6-mm full width at half maximum Gaussian kernel. Finally, using DPRSF, linear trend subtraction and temporal filtering (0.01–0.10 Hz) were performed on the time series of each voxel in order to reduce the effect of low-frequency drifts and high-frequency noise. As a quality check of fMRI date, large head motion in any direction corresponding to >3 mm or any rotation >3° would be excluded in our study.

### Harris Hip Scoring System

The Harris Hip Scale, a widely adopted approach for assessing hip function, has been demonstrated to be effective for evaluating changes in hip function, and it has been shown to have high validity, reliability, and responsiveness (Mistry et al., 2016). Consequently, we used this approach to evaluate the pain, hip function, deformity level, and motion range of ONFH patients.

### ALFF Analyses

We used the DPARSF to calculate the ALFF value, as described in previous studies (Yu et al., 2014; Golestani et al., 2017; Zhou et al., 2017). The preprocessed data were subjected to detrend and bandpass filtering (0.01–0.1 Hz). The time courses were first converted to the frequency domain *via* the fast Fourier transform algorithm. The square root of the power spectrum was computed and then averaged across 0.01–0.10 Hz at each voxel. This averaged square root was recorded as the ALFF. To reduce the global effects of variability across the participants, the ALFF of each voxel was divided by the global mean ALFF value of each subject, resulting in a relative ALFF. The global mean ALFF value was calculated for each participant within a group GM mask obtained by selecting a threshold of 0.2 on the mean GM map of all the 20 subjects. The relative ALFF value in a given voxel reflects the degree of its raw ALFF value relative to the average ALFF value of the whole brain.

### Statistical Analyses

#### Intergroup ALFF Analysis

To determine the ALFF differences between the two groups, a two-sample *t*-test was performed at each voxel (within the AAL mask and GM mask). The statistical threshold was set at |T| > 1.73 (*P* < 0.05) and the cluster size at >10 voxels (=270 mm^3^), which corresponded to a corrected *P*-value of < 0.05, and the corrections were conducted using Gaussian random field (GRF).

#### Correlation Analysis of ALFF and Harris Hip Scores

To investigate the relationship between the ALFF and functional performance in all the subjects, we computed the Pearson’s correlation coefficients between the ALFF and Harris hip scores in both the ONFH and healthy control groups combined in a voxel-wise way. The statistical threshold was set at |T| > 1.73 (*P* < 0.05) and the cluster size at > 50 voxels (=1,350 mm^3^), which corresponded to a corrected *P* of <0.05 (Wang et al., 2011).

## Results

### Demographic Characteristics and Hip Function Test

**Figure 1:**
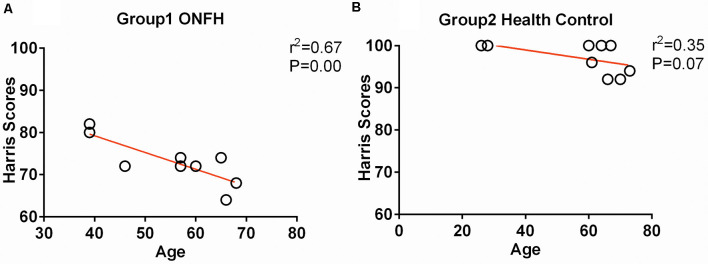
Correlation between age and Harris hip score. **(A)** The scatter plots between the Harris scores and age of the osteonecrosis of the femoral head (ONFH) participants. **(B)** The scatter plots between the Harris scores and age of healthy control participants.

**Figure 2:**
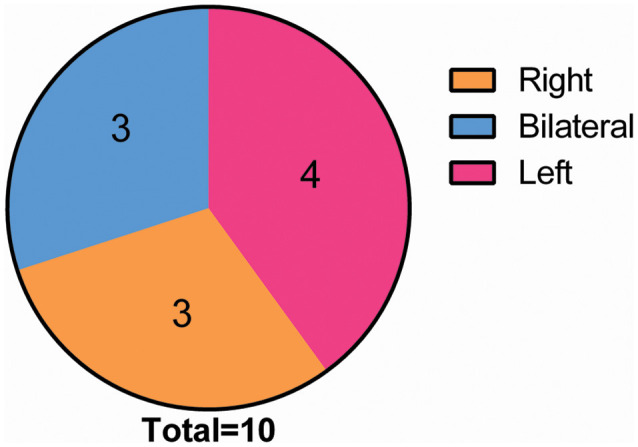
Distribution of the incidence location in the ONFH Group. Among all of the 10 ONFH patients, the condition was present in the right hip in three patients and in the left hip in four patients, and it was bilateral in three patients. Abbreviations: ONFH, osteonecrosis of the femoral head.

Eventually, after excluding subjects with excessive head movements, 10 ONFH subjects and 10 HC subjects entered the statistical analysis stage. The demographic characteristics, neuropsychological scores, and hip function scores are presented in Table 1. There were no significant differences between the two groupsin terms of sex, age, and MMSE scores; however, the Harris hip scores were significantly different between the two groups (*P* < 0.001). Figure 1 demonstrates that the correlation between age and Harris scores was significant. The differences among the affected sides were significant (*P* < 0.001) since there was a larger proportion of patients with ONFH affecting the left side in the experimental group. The distribution of sides affected with ONFH is presented in Figure 2.

### ALFF Differences Between ONFH Patients and Healthy Controls

**Figure 3:**
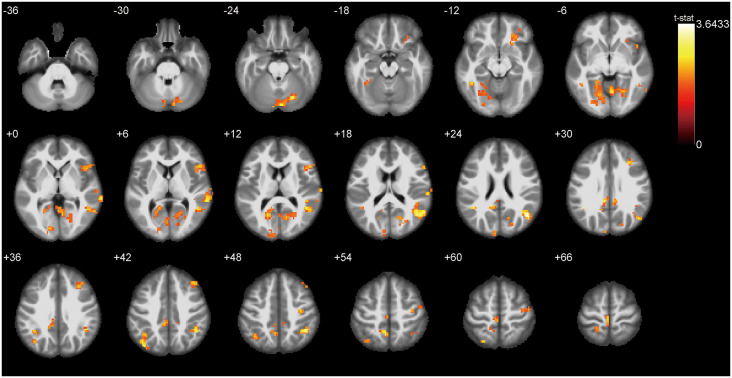
*T*-test statistical difference maps between the ONFH patients and healthy controls. There were significant ALFF differences between the two groups in the right middle occipital gyrus (MOG), right inferior parietal lobule, left angular gyri, right insula, right superior temporal gyrus, right lingual gyrus, left median cingulate and paracingulate gyri, left precuneus, left cuneus, and right parahippocampal gyrus (PHG) while differences were also observed in the right middle temporal gyrus, the left calcarine fissure, both sides of the precentral gyrus (PreCG), the bilateral postcentral gyrus (PoCG), the right triangular part of the inferior frontal gyrus (IFGtri), the right middle frontal gyrus, and the right paracingulate gyri. For the details of the regions, see Table 2. Abbreviations: ONFH, osteonecrosis of the femoral head; ALFF, amplitude of low-frequency fluctuations.

Figure 3 presents the ALFF differences between ONFH patients and healthy controls. The most significant ALFF increases in the ONFH patients were found in the right middle occipital gyrus (MOG), right inferior parietal lobule, left angular gyri, right insula, right superior temporal gyrus, right lingual gyrus, and left median cingulate and paracingulate gyri, left precuneus, left cuneus, and right parahippocampal gyrus (PHG) while increases were also observed in the right middle temporal gyrus, the left calcarine fissure and surrounding cortex, both-sides of the PreCG, the right inferior frontal gyrus, the right triangular part of the inferior frontal gyrus (IFGtri), the right middle frontal gyrus, both-sides of the postcentral gyrus (PoCG), the right anterior cingulate, and the paracingulate gyri. Significant ALFF decreases associated with ONFH were not observed.

**Table 2:** Regions exhibiting ALFF differences between the ONFH patients and healthy controls (with GRF correction).

Region	Side	MNI coordinates				
		*x*	*y*	*z*	*t*-value	Cluster size (*k*)
Middle occipital gyrus	R	48	−66	24	3.64	238
Inferior parietal	R	42	−48	45	3.45	85
Angular gyri	L	−42	−51	24	3.41	150
Insula	R	30	12	−18	3.38	29
Superior temporal gyrus	R	69	−33	3	3.25	110
Lingual gyrus	R	6	−63	−3	3.22	786
Median cingulate and paracingulate gyri	L	−9	−39	33	3.21	786
Precuneus	L	−6	−54	54	3.17	72
Cuneus	L	−6	−96	27	3.15	786
Parahippocampal gyrus	R	18	−6	−21	3.08	41
Middle temporal gyrus	R	48	−54	3	2.83	238
Calcarine fissure and surrounding cortex	L	−18	−63	12	2.74	786
Precentral gyrus	R	36	−15	51	2.71	52
Inferior frontal gyrus, triangular part	R	48	21	9	2.61	122
Middle frontal gyrus	R	30	27	30	2.60	85
Inferior temporal gyrus	L	−45	−48	−12	2.59	786
Paracentral lobule	R	0	−27	69	2.58	52
Superior parietal gyrus	L	−24	−69	60	2.56	150
Superior occipital gyrus	R	21	−78	21	2.42	786
Anterior cingulate and paracingulate gyri	R	6	51	9	2.37	14
Postcentral gyrus	L	−24	−45	69	2.41	72
Lingual gyrus	L	−21	−48	−6	2.25	786
Precentral gyrus	L	−27	−21	57	2.22	11
Fusiform	R	24	−72	−6	2.05	786

*This table shows all the local maxima separated by more than 20 mm. Regions were automatically labeled using the automated anatomical labeling (AAL) 2 atlas. x, y, and z = Montreal Neurological Institute (MNI) coordinate in the left-right, anterior-posterior, and inferior-superior dimensions, respectively. The statistical threshold was set at P < 0.05 and the cluster volume at 10 voxels between the ONFH patients and healthy controls corrected by a GRF correction. ONFH, Osteonecrosis of the femoral head; GRF, Gaussian random field; ALFF, amplitude of low-frequency fluctuations*.

**Figure 4:**
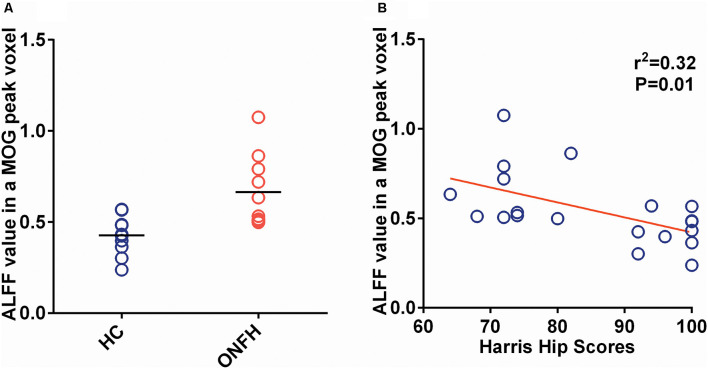
The ALFF of the MOG and Correlation of the ALFF and Harris Hip Score. **(A)** A scatter plot representing the individual MOG ALFF values in the ONFH patients and healthy controls. The group means were different between the two groups and exhibited the following trend: ALFF_ONFH_ < ALFF_HC,_ where HC represents healthy control. There were significant differences between ONFH patients and healthy controls (*t* = 3.387, *P* = 0.0033). **(B)** The scatter plot between MOG ALFF and Harris hip scores, which has a linear regression as a significant negative correlation (*r*^2^ = 0.3195, *P* = 0.0094). Abbreviations: ALFF, amplitude of low-frequency fluctuations; MOG, middle occipital gyrus; ONFH, osteonecrosis of the femoral head.

Figure 4A shows the individual MOG ALFF in ONFH patients and healthy controls. The group means were different between the two groups and exhibited a trend of ALFF_HC_ < ALFF_ONFH,_ where HC represents healthy control. There were significant differences between ONFH patients and healthy controls (*t* = 3.387, *P* = 0.0033).

### Correlations Between the ALFF and Harris Hip Scores

Figure 4B depicts the correlation maps between the ALFF and Harris hip scores in the ONFH and HC groups combined. Per the results of linear regression analysis, there were significant negative correlations (*r*^2^ = 0.3195, *P* = 0.0094) in the MOG ALFF.

## Discussion

ONFH may lead to intractable pain and the functional disability of the hip joint. Chronic pain and gait abnormality could influence the spatial patterns of intrinsic brain activity (Xie et al., 2013; Chen et al., 2015; Flodin et al., 2016; Ryan et al., 2018; Schrepf et al., 2018). Our study investigated the ONFH-related changes in the intrinsic or spontaneous brain activity by measuring the ALFF values of resting-state fMRI signals. Based on the current literature, this is the first time that a study has investigated the brain plasticity in ONFH patients. We found that there were global differences in ALFF values when comparing them between the ONFH patients and healthy controls, including in the right MOG, right inferior parietal lobule, left angular gyri, right insula, right superior temporal gyrus, right lingual gyrus, left median cingulate and paracingulate gyri, left precuneus, left cuneus, and right PHG while differences were also observed in the right middle temporal gyrus, the left calcarine fissure and surrounding cortex, both sides of the PreCG, the bilateral PoCG, the right IFGtri, the right middle frontal gyrus, and the right paracingulate gyri. Our results imply that measuring the ALFF value of intrinsic or spontaneous brain activity could be useful in characterizing the pathophysiology of ONFH.

### Sensorimotor Dysfunction

Dysfunction, including chronic pain and movement disorder of the hip joint, has long plagued ONFH patients and may lead to functional and structural plasticity in the sensorimotor system. The primary motor cortex is predominantly located in the PreCG while the primary somatosensory cortex is located in the PoCG. Previous studies have revealed that PreCG is the primary region of the motor network that is involved in planning and executing movements (Stinear et al., 2009). In clinical practice, despite the pain they experience, the major complaint of arthritis patients is regarding the impairment of motor function, and typically, the sensorimotor cortex is the main area that is analyzed (Canavero and Bonicalzi, 2013). Interestingly, in the present study, we found the enhanced activation of the bilateral PreCG and PoCG in the ONFH patients compared to in the healthy controls. It is known that patients with ONFH may continue to use the affected hip joint with an abnormal gait. The brain tries to compensate for the unusual walking posture and enhances its motor control *via* adaptive plasticity. The occipital lobe contains most of the anatomical region of the visual cortex and contributes to visual information processing and communication with the cerebral cortex (Tu et al., 2013), the increasing of ALFF in MOG also could be evidence of *via* enhancing visual-spatial abilities to assist motor control.

### Pain

Most of the previous studies have focused on the influences between fair of pain and pain perception, and the results have revealed that several brain regions, including the insular, prefrontal cortex, and anterior cingulate cortex may be involved in the development of fair of pain (Lumley et al., 2011; Lyby et al., 2011). ONFH patients had verified degrees of pain, resulting in increased ALFF values in the insula, paracingulate gyri, and IFGtri. The IFGtri is a part of the frontal gyrus of the frontal lobe. It is known that the anterior cingulate cortex is a critical hub for pain-induced mood disorders (Barthas et al., 2015). The insular cortex is critical for the perception, modulation, and chronification of pain (Lu et al., 2016). The prefrontal cortex is related to negative emotions and the descending pain inhibitory system (Maeoka et al., 2012). Based on our results, the contralateral (right-side) insula, paracingulate gyri, and IFGtri exhibited significant changes in the ALFF value. It may be inferred that ONFH-induced chronic pain could result in the functional remodeling of the brain. The pain matrix may be activated, and the pain-loop at the central level could be another adaptive plasticity response in ONFH patients, which could be an effective factor for poor clinical outcomes.

### Emotion and Cognition

Previous studies have demonstrated that chronic pain may lead to various emotional and cognitive disorders (Lyby et al., 2011). The limbic system contributes to information processing related to motivation, emotion, learning, cognition, and memory. Our results revealed increased ALFF values in the right PHG. The PHG surrounds the hippocampus and is an important part of the limbic system. The PHG plays an important role in emotion processing, center-periphery organization, high spatial frequencies, expertise, and cognition (Levy et al., 2001; Van den Stock et al., 2014). The PHG transfers major polysensory input to the hippocampus and is the recipient of different combinations of sensory information (Burwell, 2000). The enhanced ALFF value in the PHG may indicate information recoding and an integration disorder in ONFH patients (Ranganath and Ritchey, 2012). Meanwhile, the hippocampus has been considered as the brain region that contributes to processing negative emotions, such as fear and anxiety (Kajimura et al., 2015). In the present study, the obvious increase in the ALFF value of the PHG could be evidence of a compensatory reaction for abnormal emotions in ONFH patients. Some studies also elucidated the obvious abnormal resting-state activity and increase in functional connectivity of the MOG in major depressive disorder (Teng et al., 2018), it also could be evidence of a compensatory reaction for abnormal emotions in ONFH patients. Further research should be performed to investigate the psychosocial alterations associated with ONFH since a proportion of ONFH patients suffer from anxiety, depression, and other mental illnesses.

Moreover, significant ALFF decreases associated with ONFH were not observed in the present study. In all the ONFH patients, the control of the healthy side may be enhanced to adapt to the chronic pain and functional disability. Further, this may enhance the activation of extensive brain regions.

In conclusion, we have presented the changes in the ALFF value in ONFH patients using rs-fMRI. We found that the MOG exhibits the most significant group difference in terms of the ALFF. Our results have demonstrated that the abnormal spontaneous intrinsic activity of several brain regions could be the underlying pathophysiology and brain region interconnecting relation in ONFH. Furthermore, the abnormal spontaneous activity of the MOG and frontal lobe leads to several problems that should be considered. In further studies, the following steps should be taken. First, a larger sample size should be used, and sex- and age-related changes should be investigated in different sexes and ages. Second, longitudinal studies on the changes in the intrinsic or spontaneous brain should be conducted in patients with ONFH. Third, patient groups with different stages of ONFH should be enrolled for group investigations.

## Data Availability Statement

The raw data supporting the conclusions of this article will be made available by the authors, without undue reservation.

## Ethics Statement

The studies involving human participants were reviewed and approved by Medical Research Ethics Committee of Yueyang Hospital of Integrated Traditional Chinese and Western Medicine, Shanghai University of Traditional Chinese Medicine (2018-041-01). The patients/participants provided their written informed consent to participate in this study.

## Author Contributions

SF and XH performed statistical analysis and wrote the manuscript. XL, BZ, GL, and WL were involved in the fMRI scanning and data collection. BL and JX were responsible for the study design and research funding. All authors contributed to the article and approved the submitted version.

## Conflict of Interest

The authors declare that the research was conducted in the absence of any commercial or financial relationships that could be construed as a potential conflict of interest.

## Footnotes

^1^http://www.fil.ion.ucl.ac.uk/spm

## Abbreviations

ONFH: Osteonecrosis of the femoral head
rs-fMRI: Resting-state functional magnetic resonance imaging
fALFF: fractional amplitude of low-frequency fluctuation
MMSE: Mini-Mental State Examination
DPARSF: Data Processing Assistant for rsfMRI
MOG: middle occipital gyrus
PHG: parahippocampal gyrus
PreCG: precentral gyrus
IFGtri: right triangular part of the inferior frontal gyrus
PoCG: postcentral gyrus.
